# 2422. Relative Risk of Patients Having a Catheter-Associated Urinary Tract Infection Within 7 days of Having an Indwelling Urinary Catheter Reinserted Within 24 Hours of Indwelling Urinary Catheter Removal

**DOI:** 10.1093/ofid/ofad500.2041

**Published:** 2023-11-27

**Authors:** Eric Hadhazy, John Shepard, Angela Graf, Joy Goor, Bilwa Buchake, Yohan Lee, Karen McIntyre

**Affiliations:** Stanford Health Care , Palo Alto, California; Stanford University, Palo Alto, California; Stanford Health Care, Palo Alto, California; Stanford Health Care, Palo Alto, California; Stanford Health Care, Palo Alto, California; Stanford Health Care, Palo Alto, California; Stanford Healthcare, Stanford, California

## Abstract

**Background:**

Indwelling urinary catheters (IUC) are reinserted in patients who should initially be managed with intermittent straight catheterization for urinary retention. We hypothesize patients who have an IUC reinserted within 24 hours will be at higher risk of catheter-associated urinary tract infections (CAUTI) within 7 days compared to patients who were straight catheterized or did not have an IUC reinserted.

**Methods:**

A retrospective review of electronic health records (EHR) using Epic Systems (Verona, Wisconsin) was conducted for all inpatients at Stanford Hospital, Palo Alto, CA who had an IUC removed and a CAUTI defined by the National Healthcare Safety Network (NHSN) between January 1, 2020 to March 31, 2023. Patients with an IUC reinserted within 24 hours from initial removal of an IUC were compared to patients who had an IUC removed and did not have a reinsertion of an IUC within 24 hours. Relative risk of a CAUTI was the primary outcome metric.

**Results:**

Between January 1, 2020 and March 31, 2023 there were 30,161 IUCs removed and 204 NHSN defined CAUTI identified within 7 days of removal that were included in the study. Patients who had an IUC reinserted within 24 hours had significant risk (RR: 7.96, p< 0.05) of having a CAUTI within 7 days post IUC removal compared to patients who did not have an IUC reinserted within 24 hours.

**Figure 1.**
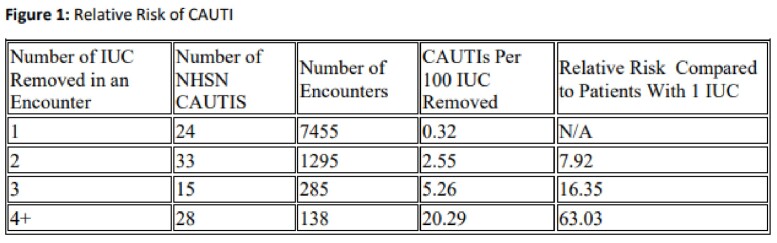
Relative Risk of CAUTI with Indwelling Catheter Reinsertion

**Conclusion:**

Reinsertion of IUCs in patients with urinary retention following IUC removal should be managed with intermitted straight catheterization or more conversative methods if possible. Patients who are re-catheterized with an IUC within 24 hours of removal are at significantly higher risk of developing a CAUTI within 7 days compared to patients who are straight catheterized or do not have a Foley catheter reinserted. Healthcare providers should use alternative methods of bladder management to improve patient outcomes.

**Disclosures:**

**All Authors**: No reported disclosures

